# Reservoir hosts for *Gyrodactylus salaris* may play a more significant role in epidemics than previously thought

**DOI:** 10.1186/s13071-014-0576-5

**Published:** 2014-12-20

**Authors:** Giuseppe Paladini, Haakon Hansen, Chris F Williams, Nick GH Taylor, Olga L Rubio-Mejía, Scott J Denholm, Sigurd Hytterød, James E Bron, Andrew P Shinn

**Affiliations:** Institute of Aquaculture, School of Natural Sciences, University of Stirling, Stirling, FK9 4LA Scotland UK; Norwegian Veterinary Institute, Section for Parasitology, P.O. Box 750, Sentrum, NO-0106 Oslo Norway; Environment Agency, National Fisheries Laboratory, Bromholme Lane, Brampton, PE28 4NE UK; Centre for Environment, Fisheries & Aquaculture (Cefas), Weymouth Laboratory, Barrack Road, Weymouth, DT4 8UB UK; Animal Breeding and Genomics, Animal & Veterinary Sciences, SRUC, Roslin Institute Building, Easter Bush, Midlothian, EH25 9RG UK; Fish Vet Group Asia Ltd., 99/386, Chaengwattana Building, Chaengwattana Rd., Kwaeng Toongsonghong, Khet Laksi, Bangkok 10210 Thailand

**Keywords:** Parasite, *Salmo salar*, Atlantic salmon, *Salmo trutta*, Brown trout, *Thymallus thymallus*, Grayling, Susceptibility, Monogenea, Pathogen

## Abstract

**Background:**

*Gyrodactylus salaris* Malmberg, 1957 has had a devastating impact on wild Norwegian stocks of Atlantic salmon *Salmo salar* L., and it is the only Office International des Epizooties (OIE) listed parasitic pathogen of fish. The UK is presently recognised as *G. salaris*-free, and management plans for its containment and control are currently based on Scandinavian studies. The current study investigates the susceptibility of British salmonids to *G. salaris*, and determines whether, given the host isolation since the last glaciation and potential genetic differences, the populations under test would exhibit different levels of susceptibility, as illustrated by the parasite infection trajectory over time, from their Scandinavian counterparts.

**Methods:**

Populations of *S. salar*, brown trout *Salmo trutta* L., and grayling *Thymallus thymallus* (L.), raised from wild stock in UK government hatcheries, were flown to Norway and experimentally challenged with a known pathogenic strain of *G. salaris*. Each fish was lightly anaesthetised and marked with a unique tattoo for individual parasite counting. A single Norwegian population of *S. salar* from the River Lærdalselva was used as a control. Parasite numbers were assessed every seven days until day 48 and then every 14 days.

**Results:**

*Gyrodactylus salaris* regularly leads to high mortalities on infected juveniles *S. salar*. The number of *G. salaris* on British *S. salar* rose exponentially until the experiment was terminated at 33 days due to fish welfare concerns. The numbers of parasites on *S. trutta* and *T. thymallus* increased sharply, reaching a peak of infection on days 12 and 19 post-infection respectively, before declining to a constant low level of infection until the termination of the experiment at 110 days.

**Conclusions:**

The ability of *S. trutta* and *T. thymallus* to carry an infection for long periods increases the window of exposure for these two hosts and the potential transfer of *G. salaris* to other susceptible hosts. This study demonstrates that *G. salaris* can persist on *S. trutta* for longer periods than previously thought, and that the role that *S. trutta* could play in disseminating *G. salaris* needs to be considered carefully and factored into management plans and epidemics across Europe.

## Background

There are over 430 described species of *Gyrodactylus*, small ectoparasitic monogenean worms principally infecting fish [1]. While most species of *Gyrodactylus* cause relatively little harm to their hosts, other species such as *Gyrodactylus salaris* Malmberg, 1957 which is an OIE (Office International des Epizooties) listed pathogen, has had a notable catastrophic impact on Norwegian populations of juvenile Atlantic salmon, *Salmo salar* L. [2,3]. This parasite species was introduced to Norway on at least three separate occasions [4] and to date has been recorded from 49 Norwegian rivers, and the effects on the *S. salar* populations have necessitated extreme measures to control the parasite. These include the use of the biocide rotenone to kill-out complete river systems in order to remove the entire resident fish and, consequentially, the *G. salaris* population [3,5]. Given the impact that *G. salaris* has had in Scandinavia and Russia [6-9], Norway and the UK have surveillance programmes screening species of native salmonids that may serve as potential hosts for *G. salaris*. In the UK, this includes monitoring resident populations of potential hosts such as brown trout *Salmo trutta* L., Arctic charr *Salvelinus alpinus* (L.), grayling *Thymallus thymallus* (L.) and *S. salar*, whilst in Norway farmed rainbow trout *Oncorhynchus mykiss* (Walbaum) and both farmed and wild *S. salar* populations are screened.

The reports of *G. salaris* in Poland [10] and Italy [11] purportedly linked to the movement of salmonid stocks across borders emphasise the biosecurity risk this pathogen poses to countries with resident salmonid populations.

Great Britain and Northern Ireland (forming the United Kingdom), and some selected watersheds in Finland, are currently recognised *G. salaris*-free following the studies of Platten *et al*. [12], Shinn *et al*. [13], and on-going government-based surveillance programmes (www.scotland.gov.uk for Scotland; www.cefas.defra.gov.uk for England and Wales). Given the value of the UK’s recreational *S. salar* and *S. trutta* fishing, which is worth in excess of £350 million [14], it is important that the UK’s *G. salaris*-free status is upheld. Coarse and game angling figures for Scotland in 2010 were estimated at over £100 million (www.scotland.gov.uk), whilst recreational and commercial *S. salar* and *S. trutta* fisheries in England and Wales in 2001 (last figures available) had a capital value of £130 million (www.cefas.defra.gov.uk).

Other than *S. salar*, *G. salaris* has been demonstrated to colonise and reproduce on a large number of salmonids [15-21], and, under experimental studies, on a number of non-salmonid species as well [22-25]. The lack of clinical signs of disease on some of these hosts may allow *G. salaris* infections to go undetected. This is well demonstrated by the study of Paladini *et al*. [11], where the examination of formalin-preserved material in farm archives indicated that *G. salaris* had been in Italy on *O. mykiss* for at least nine years prior to discovery. Such asymptomatic hosts may represent a serious problem in that they can serve as reservoirs playing an important role in the epidemiology and dispersal of *G. salaris* across Europe [26-28].

Existing UK dispersion models [29] and management plans for the containment of *G. salaris* are based on the assumption that in the worst-case scenario, British stocks of *S. salar* would be vulnerable to *G. salaris* and therefore at risk [30,31], as would all the salmonids within an area occupied by *S. salar*, which may act as reservoir hosts. No assumptions are made for other salmonids in the UK. Although *S. trutta* have a widespread distribution throughout England and Wales, it is mainly towards the west of the country where their occurrence overlaps with the natural habitat range of wild *S. salar*. Likewise, many *O. mykiss* aquaculture sites in the UK are in close proximity to resident *S. trutta* populations; it is assumed that British populations of *S. trutta* would be entirely resistant to *G. salaris* infection and unaffected [15,32,33]. The natural distribution of *T. thymallus* populations in the UK overlaps with both *S. salar* and *S. trutta*. From previous studies, it is suggested that *T. thymallus* would be relatively resistant to *G. salaris* infection, although it has been demonstrated that the parasite can survive and reproduce on Scandinavian *T. thymallus* for 143 days [16,34].

Following models determined for Scandinavian populations of *S. trutta* and *T. thymallus* [3], these hosts are thought to harbour low-level infections of *G. salaris* for a few weeks, not displaying the exponential increase in numbers seen on *S. salar*, although they may act as reservoirs of infection. Native UK stocks of *S. trutta* and *T. thymallus*, however, have been separated from their Scandinavian counterparts since the last period of glaciation [35-37], and their relative patterns of susceptibility and/or resistance may therefore differ from those predicted from Norwegian studies. Assumptions that UK *S. salar* are susceptible to *G. salaris* are derived from a few earlier studies which tested the susceptibility of two Scottish populations of *S. salar* (*i.e.* from the Rivers Shin and Conon) to *G. salaris* originating from the River Figga, county Nord-Trøndelag, Norway [31,38,39]. The experimental exposure of other British salmonids (*i.e. S. trutta*, *T. thymallus*, *etc*.) to *G. salaris* has not been conducted to date.

The current study determines for the first time the responses of different English and Welsh salmonids to a pathogenic strain of *G. salaris* and sets out to make a contribution, not only to existing UK national *G. salaris* management planning, but also to disease risk management across Europe. The findings from this study highlight the importance of reservoir hosts in the risk of establishment and spread of *G. salaris*, increasing its rapid spread and placing greater pressure on parasite detection and management approaches. The study also discusses the extent to which laboratory conditions might affect the results of infection experiments, and gauge whether extrapolation from existing results is appropriate for UK management policy.

## Methods

All experimental procedures and husbandry practices involving animals were conducted in compliance with the Animals Scientific Procedures Act 1986 (Home Office Code of Practice. HMSO: London, January 1997), in accordance with EU regulation (EC Directive 86/609/EEC), and approved by the Animal Ethics and Welfare Committee of the University of Stirling, UK. The experimental work was also approved by the Norwegian Animal Research Authority (NARA).

### Origin of experimental salmonid populations

Populations of *S. salar*, *S. trutta* and *T. thymallus* supplied for this study were obtained from registered Environment Agency fish farms located in England and Wales. Wild adult broodstock of each species were captured by means of electrofishing and transported live to hatchery facilities. The fish were subsequently held, stripped of eggs and milt, and the off-spring reared under the conditions described. This work was authorised and conducted by staff of the Environment Agency under the statutory duty to maintain, improve and develop fisheries in England and Wales.

### *i) Salmo salar* from the River Dee, Wales

In 2010/2011, eggs from wild *S. salar* caught in the River Dee, northern Wales, were stripped, fertilised and reared to 0+ parr in the Environment Agency’s (EA) Maerdy Hatchery, Corwen, Conwy, Wales (52°59’18.18” N; 3°27’48.18” W). The eggs began hatching around mid-January 2011. The fish were reared on ambient water (av. 2.7°C) from the Afon Ceirw using a natural photoperiod regime and a 1% body wt day^−1^ daily feed ration (Skretting Nutra Parr 02). The fish had a mean total length of 67.0 ± 0.2 mm and a mean weight of 3.4 ± 0.3 g at the time they were transported to Norway.

### *ii) Salmo trutta* from the River Tyne, England

In November 2010, adult *S. trutta* broodstock were collected from the River Rede, a tributary of the River Tyne, Northumberland, England. The ripe female fish were stripped and fertilised, and the eggs maintained at the EA’s Kielder Hatchery (55°14’00.45” N; 2°34’39.69” W). Egg hatching occurred over the period March to April 2011. The eggs and juvenile fish were maintained at ambient water temperatures (0–18.5°C), with natural photoperiod conditions and a 0.1–2.8% body wt day^−1^ daily feed ration (Skretting Emerald Fry 00, 01 and 02 crumb) over a period of 303–316 days, until they were transported to Norway. The 0+ parr had a mean total length of 85.2 ± 0.5 mm and a mean weight of 4.45 ± 0.4 g at the time of transportation.

### *iii) Thymallus thymallus* from the River Nidd, England

*Thymallus thymallus* broodstock originating from the River Nidd, Knaresborough, England were stripped and the eggs reared in the EA’s Calverton Fish Farm (53°02’01.43” N; 1°03’05.95” W). Egg hatching began in April 2011. The fish were reared on borehole water (mean 10 ± 1°C) and a constant natural photoperiod (05.00-21.30 without adjustment). First *ad libitum* feed was *Artemia salina* for approximately two weeks, followed by a gradual weaning onto Coppens TroCo Crumble Top and Crumble HE feed. Throughout the rearing phase, the dried diet was supplemented by gamma-radiated chironomids. The 0+ *T. thymallus* had a mean total length of 111.7 ± 0.7 mm and weight 12.7 ± 0.5 g at the time of their transportation to Norway in January 2012.

### *iv) Salmo salar* from the River Lærdalselva, Norway, control group

The *S. salar* juveniles (age 0+, mean total length 90.5 ± 0.5 mm, mean weight 5.5 ± 0.5 g) used as a control for this trial originated from the River Lærdalselva (61°02’ N; 7°36’ W), western Norway, and were obtained from the Ljøsne hatchery, near Lærdal. The fish were reared on ground water, heated to 11°C during the first week post-hatching, and subsequently on heated ground water at 9°C for the rest of the culture period until their transfer to the Norwegian Veterinary Institute (NVI) in Oslo. Fish were fed *ad libitum* with a commercial pellet diet (Skretting Nutra Plus 01). The fish were transported to the NVI in oxygenated water, using the same methodology described in the section below (“Transportation of salmonids to Norway”) to ensure similar conditions for both control and experimental fish. Upon arrival at the fish holding department, the fish were immediately transferred to a holding tank (60 × 60 × 70 cm) and acclimated in laboratory water at 11°C for seven days prior to the start of the experiment. A single tank of 10 fish was used during the trial.

### Transportation of salmonids to Norway

In January 2012, 70 *S. salar* originating from the Welsh River Dee, 70 *S. trutta* from the English River Tyne, and 70 *T. thymallus* from the English River Nidd were transported from Environment Agency fish farms to the secure research aquarium facility within the NVI in Oslo. Each population of fish was prepared by EA staff at the hatchery, by double-bagging the fish in oxygenated polyethylene bags and placing them on chill packs, to ensure a stable temperature during transportation. These were sealed in International Air Transport Association (IATA)-approved robust polystyrene boxes, each of which measured 65 cm (depth) × 58 cm (length) × 49 cm (width). The polystyrene boxes were then placed inside a double-walled cardboard box to ensure protection during transportation. The relevant permissions from the Chief Veterinary Officer in the UK and in Norway, from the Norwegian authorities (The Directorate for Nature Management and the Food Safety Authorities) and from the NVI, were obtained before the fish were shipped. The project was also monitored by senior government officials and fish biologists within Defra (Department of the Environment, Fisheries and Rural Affairs), London; the EA (Environment Agency), National Fisheries Laboratory, Brampton; and, at Cefas (Centre for Environment, Fisheries & Aquaculture Science), Weymouth Laboratory, UK. The fish were transported using the specialist live animals courier, Gulf Agency Company (GAC) Logistics, through Manchester International airport to Gardermoen Airport, Oslo, Norway. Once a visual health inspection of the fish and their welfare by the on-duty veterinary surgeon at Oslo had been made and were cleared as fit to continue their onward journey, the fish were transported immediately to the NVI, Oslo research facility. The fish, still within their plastic bags, were transferred to 0.6 m (diameter) × 0.7 m (depth) fibreglass tanks supplied with a constant 11 ± 1°C water flow rate of 200 ml min^−1^ and additional aeration; the temperature of the water in the bags was allowed to adjust to that of the tank, before the bags were opened and the fish released. No fish were lost during the 6 h transportation exercise, *i.e.* from the time they were packed to the time they were released in their tanks at NVI. The fish were left to acclimate for a further seven days before the infection trial was started. The source of the water used within the aquarium was from the Oslo city domestic supply, which passes through a particle filter (Structural C-2160-F7 composite, 310 L) and an activated carbon filter (GAK 170) prior to use.

### Source of *Gyrodactylus salaris* used for the trial

The *G. salaris* strain used in the experiment, *i.e.* mitochondrial haplotype A [4], was obtained from wild *S. salar* juveniles, sampled by electrofishing in the River Fusta, northern Norway (65°54'9.57"N 13° 9'50.92"E). This particular strain of *G. salaris* has previously been tested experimentally and shown to be pathogenic to *S. salar* [40].

### *Gyrodactylus salaris* infection procedure

Thirty fish from each population were randomly selected, using the simple random sample (SRS) method [41], and then infected by transferring them to a static 30 L tank with aeration into which approximately 3,000 *G. salaris* had been added by gently scraping the excised fins of heavily infected aquarium-held fish. This approach has been used effectively in the past [16,32,42] to ensure an infection of 50–80 parasites fish^−1^ over a 24 h exposure period, and it assumes that 50% of parasites will successfully transfer to the new host. Following the exposure period, each fish, which was tattooed with a unique mark using alcian blue (40 mg ml^−1^), was lightly anaesthetised in metacain Finquel® Vet. (50 mg L^−1^), and the total number of *G. salaris* on each fin and body zone was counted under a Leica MZ7.5 dissecting microscope at × 4 magnification. This anaesthetic has also been used in previous experiments [43,44], as it does not affect *Gyrodactylus* survival. Alcian blue marking was preferred as a rapid, reliable, easy, and long-lasting method [45], rather than fin clipping, as fins are the preferred microhabitat of *G. salaris* [3].

Each fish was then randomly assigned to one of three experimental tanks (10 L circular; flow-through 200 ml min^−1^). Each population was tested in triplicate (each replicate n = 10 juvenile fish), with the exception of the River Lærdalselva Norwegian *S. salar* control, which was already a standardised model previously tested in several trials by the same research aquarium, and for which only a single tank of 10 fish was infected. The *S. trutta* population, however, was highly aggressive when separated into the three small tanks of 10 fish each. For this reason, a single 0.6 × 0.7 m fibreglass tank (30 L; flow-through 200 ml min^−1^) containing all 30 fish was used for the *S. trutta* trial.

Seven days later, each tank of fish was anaesthetised and the number of *G. salaris* on each individually marked fish was determined by manual counting parasites with the aid of a Leica MZ7.5 stereo-microscope. The fish were sampled approximately every seven days thereafter until day 48 and then every 14 days. The fish were fed with a commercial pelleted diet (Skretting Nutra Parr 1.8) once a week.

## Results

**Figure 1:**
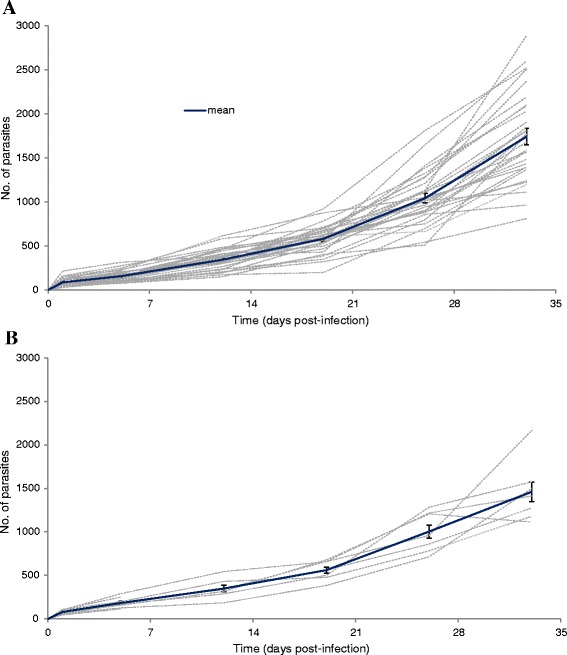
***Gyrodactylus salaris*** **Malmberg, 1957 on two strains of Atlantic salmon** ***Salmo salar*** **L.** Experimental infection of *G. salaris* (Fusta strain, haplotype A) on **(A)** *S. salar* (n = 30; three replicates of 10 fish each), from the River Dee in Wales, UK; and **(B)** the control group of *S. salar* (n = 10) from the River Lærdalselva, Norway. The growth on the two hosts (Welsh and Norwegian *S. salar* populations) is shown on the same scale for direct comparison. Grey dotted lines represent the number of parasites assessed on each individually marked fish; mean intensity of infection is shown in blue line, including standard error of the mean (SEM).

**Figure 2:**
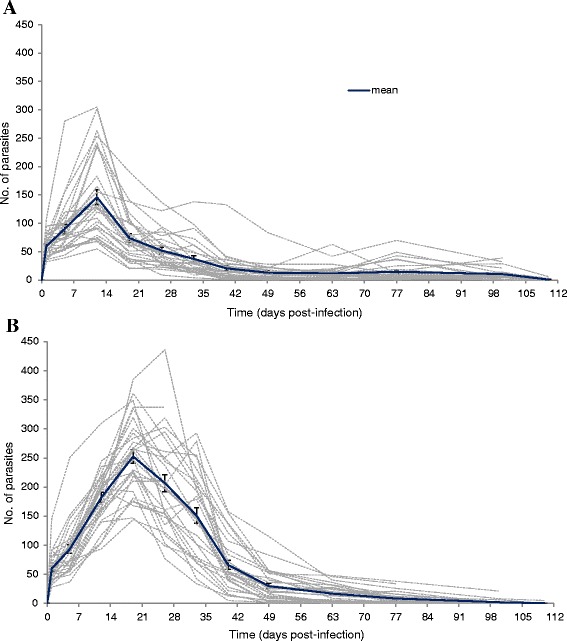
***Gyrodactylus salaris*** **Malmberg, 1957 on brown trout** ***Salmo trutta*** **L. and grayling** ***Thymallus thymallus*** **(L.).** Experimental infection of *G. salaris* (Fusta strain, haplotype A) on a population of **(A)** *S. trutta* (n = 30), from the River Tyne in England, UK; and **(B)** *T. thymallus* (n = 30; three replicates of 10 fish each), from the River Nidd in England, UK. The growth on *S. trutta* and *T. thymallus* is shown on the same scale for direct comparison. Grey dotted lines represent the number of parasites assessed on each individually marked fish; mean intensity of infection is shown in blue line, including standard error of the mean (SEM).

The dynamics of *G. salaris* infection on each of the three salmonid populations originating from England and Wales were compared against an infection of *G. salaris* on Norwegian *S. salar* over trials lasting up to 110 days. The parasite numbers on each individually marked fish and the mean of each replicate are shown in Figures 1 and 2, while the mean parasite burden and the range for each population of fish, at each sampling time point, are presented in Table 1. The initial *G. salaris* infection burdens, 24 h post-infection (p.i.), were: 87.0 parasites fish^−1^ (28–215) on the Welsh *S. salar* from the River Dee; 79.6 parasites fish^−1^ (46–108) on the Norwegian control; 59.7 parasites fish^−1^ (32–107) on *S. trutta*; and, 59.8 parasites fish^−1^ (28–146) on *T. thymallus* (see Table 1).

**Table 1:** **Intensity of** ***Gyrodactylus salaris*** **Malmberg, 1957 infection on** ***Salmo salar*** **L. from the River Dee, Wales and from the Laerdalselva, Norway (control group), from** ***Salmo trutta*** **L. from the River Tyne, England and from** ***Thymallus thymallus*** **(L.) from the River Nidd, England**

**Time points (days)**	***Salmo salar***	***Salmo salar*** **(control)**	***Salmo trutta***	***Thymallus thymallus***
	**(R. Dee, Wales)**	**Laerdalselva, Norway**	**(R. Tyne, England)**	**(R. Nidd, England)**
1	87.0 ± 8.2 (28–215)	79.6 ± 6.6 (46–108)	59.7 ± 3.5 (32–107)	59.8 ± 4.3 (28–146)
5	157.4 ± 11.2 (76–314)	183.4 ± 17.0 (114–291)	90.1 ± 8.5 (39–280)	93.4 ± 7.5 (37–251)
12	343.6 ± 21.3 (151–615)	349.1 ± 35.8 (184–544)^a^	145.9 ± 13.4 (55–305)	182.5 ± 8.4 (94–310)
19	581.6 ± 28.6 (200–923)	560.4 ± 35.3 (385–679)	74.0 ± 7.2 (20–191)	252.6 ± 11.7 (144–385)
26	1043.5 ± 54.1 (511–1812)	1003.1 ± 73.2 (714–1284)	52.0 ± 5.6 (9–137)	206.7 ± 14.8 (77–436)
33	1741.5 ± 93.1 (810–2890)	1459.7 ± 111.5 (1114–2165)	37.3 ± 5.5 (4–138)	151.1 ± 13.3 (34–293)^d^
40	-	-	20.7 ± 4.4 (1–133)	66.1 ± 7.8 (5–158)
49	-	-	13.1 ± 2.8 (0–84)	29.7 ± 4.9 (0–115)
63	-	-	11.8 ± 2.4 (0–63)	16.9 ± 2.7 (0–48)
77	-	-	14.5 ± 3.0 (0–70)^b^	8.5 ± 1.9 (0–38)^e^
100	-	-	10.6 ± 1.8 (0–39)^c^	2.1 ± 0.8 (0–21)^f^
110	-	-	0.9 ± 0.3 (0–6)	0.3 ± 0.2 (0–5)

Footnotes: Fish mortalities throughout the duration of the experiment. Parasite numbers were assessed on dead individuals.

Control *S. salar*: ^a^three dead fish not linked with *G. salaris* infection.

*Salmo trutta*: ^b^five dead fish due to power outage and temporary cessation in water flow on day 69; ^c^further two dead fish due to the stress derived by the previous power outage.

*Thymallus thymallus*: ^d^one dead fish; ^e^further five dead fish due to power outage and temporary cessation in water flow on day 69; ^f^further six dead fish due to the stress derived by the previous power outage.

The mean intensity ± standard error of the mean (SEM) and the range in parentheses are presented for each time point post-infection (p.i.) and host.

The results obtained demonstrate that the Welsh *S. salar* are highly susceptible to *G. salaris* infection (mean intensity ~1,742 parasites fish^−1^ in 33 d; see Figure 1A), when compared against the Norwegian control tank of fish which had a mean infection intensity of ~1,460 parasites fish^−1^ over the same time period (Figure 1B). These fish were unable to initiate a successful defence against the parasite and the experiment was terminated on day 33 p.i. due to concerns for fish welfare, following the classical initial symptoms of parasitic disease, *e.g.* flashing, eroded fins, increased mucus production giving the fish a grey colouration.

The infections of *G. salaris* on the *S. trutta* from the River Tyne peaked after ~12 days (mean intensity 145.9 parasites fish^−1^; Figure 2A), whilst those on the River Nidd *T. thymallus* peaked after ~19 days (mean intensity 252.6 parasites fish^−1^; Figure 2B). Thereafter, the extent of parasite infection decreased on both hosts. The *G. salaris* infection had almost disappeared on both sets of fish by the time the experiment was terminated on day 110 p.i. The population of *G. salaris* on three of the 30 *T. thymallus* that were tested appeared to display two peaks of infection on days 19 (av. 238.0 ± 49.4 parasites fish^−1^) and 33 (av. 250.3 ± 62.2 parasites fish^−1^) p.i., with a subsequent steady decrease in parasite numbers from day 26 p.i. until the experiment was terminated on day 110 p.i. *Salmo trutta* showed a similar response, with three *S. trutta* displaying two peaks of infection on days 12 (av. 119.3 ± 14.2 parasites fish^−1^) and 26 (av. 83.0 ± 10.1 parasites fish^−1^) p.i., with a subsequent steady decrease in numbers from day 19 p.i. onwards. By day 110 p.i., the infection on most fish had disappeared; only seven of the *S. trutta* were still infected (range 1–6 parasites fish^−1^; see Figure 2A), and only two of the 30 *T. thymallus* were infected (*i.e.* one with one parasite, the other with five *G. salaris*; see Figure 2B).

The experiment was terminated on day 110 p.i. out of welfare concerns for the fish and that sufficient data had been collected to inform the likely response of these populations of fish to *G. salaris* (haplotype A) infection. Prolonging the infection was unlikely to result in additional information and would incur additional unnecessary operational costs.

A power outage on day 69 p.i., which resulted in an overnight temporary cessation in water flow to the *T. thymallus* and *S. trutta* tanks, resulted in the loss of five *S. trutta* and five *T. thymallus*. In the following two parasite counts, an increase in parasite number was observed on 23 of the 30 *S. trutta* and on two of the 30 *T. thymallus*. The parasites on the dead fish were counted immediately on the discovery of the fish mortalities, and were included in the total count. After the power outage, the number of parasites on each of the five dead *S. trutta* were counted following their removal. Only the parasite number on one fish had increased (*i.e.* from one to eight *G. salaris* fish^−1^), whilst on the other four fish the number had decreased (*i.e.* from 63 to 12; from 28 to 11; from 17 to two; and from 16 to two *G. salaris* fish^−1^). The number of *G. salaris* on the dead *T. thymallus* were also determined and in each case the number of parasites had decreased.

The remaining pools of fish from the Rivers Dee (n = 40), Lærdalselva (n = 10), Nidd (n = 40) and Tyne (n = 10) that were brought in for the experiment but not subjected to experimental infection were maintained in separate tanks throughout the duration of the experimental trial. There were no mortalities in these tanks of fish over the 110 day trial period, and were confirmed free of an existing *Gyrodactylus* infection by visual examination.

**Figure 3:**
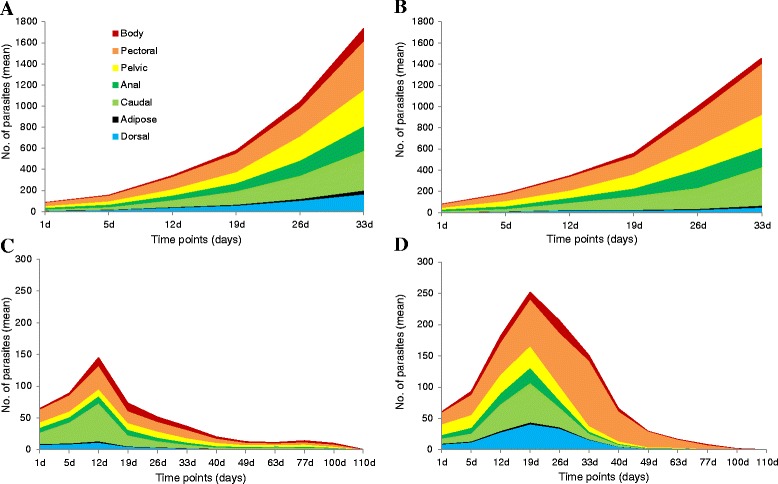
**Distribution of** ***Gyrodactylus salaris*** **Malmberg, 1957 on the fins and body of the four salmonids.** The distribution of *G. salaris* (Fusta strain, haplotype **A)** on the fins and body of a population of: **(A)** Atlantic salmon *Salmo salar* L. from the River Dee, Wales, UK (n = 30); **(B)** the *S. salar* control from the River Lærdalselva, Norway (n = 10); **(C)** brown trout *Salmo trutta* L. from the River Tyne, England, UK (n = 30); and **(D)** grayling *Thymallus thymallus* (L.) from the River Nidd, England, UK (n = 30). The growth on the two *S. salar* populations, and on *S. trutta* and *T. thymallus* is shown on the same scale for direct comparison.

Figure 3 shows the average distribution of *G. salaris* across the body and fins of each fish species throughout the experimental infection, and the graphs illustrate the importance of the fins as the preferred site of infection. The distribution of *G. salaris* on the two *S. salar* populations (Figure 3A–B) suggests that the pectoral, caudal and pelvic fins are the preferred sites of colonisation, where the parasites are evenly distributed over time. The number of *G. salaris* on the Welsh *S. salar* was also seen to increase on the eyes throughout the duration of the trial, although not given as a specific category in Figure 3A. The distribution of *G. salaris* on *S. trutta* and *T. thymallus* indicates that parasites have a preference towards occupying the caudal fin during the first 19 days of infection, and following this period, pectoral fins are the preferred site of colonisation on both hosts (Figure 3C–D). After 19 days, the parasite numbers on *S. trutta* increased also on the pelvic fins, whilst the third most colonised body part for *T. thymallus* was the dorsal fin (Figure 3C–D).

## Discussion

The infection of Welsh *S. salar* from the River Dee followed the expected infection trajectory with fish being highly susceptible to *G. salaris* infection. The trial found infections rapidly rose to ~1,742 parasites per fish in just 33 days. This finding is in close agreement with the response of *S. salar* (Atlantic strain) populations from elsewhere, including those tested from Scotland [31].

The increasing number of *G. salaris* observed on the eyes of the Welsh *S. salar* may be explained in that the eye represents an immunologically-privileged site [46,47] and therefore the immune response to parasitic infection is believed to be lower on this organ [48]. The observed increase in the number of parasites may reflect parasites moving away from skin and fin sites to avoid the host’s immune response, as has been suggested by other researchers [49-51].

The River Tyne *S. trutta* and the River Nidd *T. thymallus* were both responsive to *G. salaris* infection, with parasite numbers increasing and then subsequently declining to near extinction over the 110 days the trial was run.

Differences in the mean length of fish between species may affect parasite infection dynamics, as shown in other gyrodactylid model systems [52,53]. Although every attempt was made to provide similar sized fish from the available stocks for the start of the experimental trial and although there were differences in the length and weights of each host, the infection protocol resulted in similar starting infections (see Figure 2A–B; Table 1). The intra-variability in *G. salaris* infections shown by *S. trutta* and *T. thymallus* may be due to different susceptibilities within the same host species [54]. The different initial infection numbers seen on the four hosts may be due to the individual behaviour of the fish during the infection period, although all the fish were treated the same. There were no mortalities as a direct result of parasitic infection and there was no discernible change in fish behaviour. The *S. trutta* population was initially divided into three small groups of 10 fish each, but soon after the split, the fish showed highly aggressive behaviour causing losses of half of the population. The aggressive behaviour, including biting, chasing and fighting, in juvenile *S. trutta* has been previously documented [55] as a consequence of territoriality and competition for food in confined spaces. It is not clear, however, why aggression levels in the current trial increased dramatically with fish being split up into smaller groups, since the fish densities in the three small tanks with 10 fish each and in the single large tank containing 30 fish were the same. The lack of habitat enrichment within the tanks, *i.e.* features within which fish can hide or avoid aggression, may be a causal factor independent of stocking density.

The distribution of *G. salaris* on *S. trutta* and *T. thymallus* changed over time from the caudal fin in the first three weeks post infection, to the pectoral fins thereafter (Figure 3C–D). The shift in host sites may be a result from localised host immune reactions induced by the parasites [51,56].

Five *S. trutta* and five *T. thymallus* were lost due to a power outage on day 69 p.i., which temporarily stopped the water flow in the tanks. The stress imposed on the remaining fish, coupled with the possibility of parasite transfer from dead hosts due to fish cannibalism [57], are possible explanations for the observed small increase in parasite numbers on day 77 p.i. There was no marked variance, however, in parasite numbers between dead and live fish, therefore the average *G. salaris* intensity of infection on *S. trutta* and *T. thymallus* in the following counts are not considered to be due to parasites moving off the dead fish and colonising new hosts.

Although every precaution was taken to ensure fish welfare was upheld throughout the duration of the susceptibility trial, the level of stress placed upon each population of fish during their transportation from the UK to Norway and in their experimental tanks is not known. Whilst the 110 day period of infection may not accurately reflect how British populations of *S. trutta* in the wild would respond to *G. salaris*, if introduced into the UK, the trial has shown that the River Tyne population of *S. trutta* are able to manage infections and keep numbers to a low level, even under periods of anticipated stress. Although there were no *G. salaris*-related *S. trutta* or *T. thymallus* mortalities, the concern is that populations of these two species under stress may extend the period over which individuals can carry an infection of *G. salaris*, therefore, increasing the possible risk of parasite transfer to other fish species. Currently, 18 *G. salaris* mitochondrial haplotypes (strains) have been identified by cytochrome oxidase I (COI) analysis [4,11,58-64]. Most of the previous experimental findings (see Table 2), however, are based on studies using *G. salaris* “originating” from the River Lierelva (Norway), *i.e.* haplotype F according to the study of Hansen *et al*. [4]. This haplotype has been commonly found on *O. mykiss* and *S. salar* [4,58], and is also found on Arctic charr [64]. The study conducted by Bakke and MacKenzie [31] on Scottish *S. salar*, however, used a strain of *G. salaris* originating from the River Figga, Norway (Table 2), most likely corresponding to haplotype A (though not stated, this is interpreted from the map of haplotype distribution presented in Hansen *et al*. [4]). The strain of *G. salaris* used in the current study was derived from the River Fusta in the Vefsna region of Norway and corresponds to haplotype A, which has been demonstrated to be pathogenic to a Norwegian strain of *S. salar* [40].

**Table 2:** ***Gyrodactylus salaris*** **Malmberg, 1957 haplotypes used in previous experiments ascertaining the susceptibility of different strains of** ***Salmo salar*** **L. (A = Atlantic strain; B = Baltic strain)**

**Reference**	**Origin of** ***S. salar*** **tested in previous studies (rivers)**	**Origin of the** ***G. salaris*** **strain**	***G. salaris*** **haplotype**	**Parasite population dynamics**	**Host response** ^**1**^
Bakke [53]	A: Alta, Lone, Drammenselva and Lierelva (Norway)	R. Drammenselva	F	exponential growth	susceptible
	B: Neva (Russia)	R. Drammenselva	F	declining after 3 weeks	responding
Bakke and MacKenzie [31]	A: Conon and Shin (Scotland) and Lierelva (Norway)	R. Figga	A*	exponential growth	susceptible
Bakke *et al*. [30]	A: Alta and Lone (Norway)	R. Drammenselva	F	exponential growth	susceptible
	B: Neva (Russia)	R. Drammenselva	F	declining after 3 weeks	innately resistant and responding
Bakke *et al*. [65]	A: Akerselva (Norway)	unknown	unknown	exponential growth	susceptible
Bakke *et al*. [32]	A: Alta (Norway)	R. Lierelva	F	exponential growth	susceptible
	♀A × ♂ *Salmo trutta* hybrids: Alta (Norway) × Fossbekk (Norway)	R. Lierelva	F	declining after 3 weeks	innately resistant and susceptible
	♂A × ♀ *S. trutta* hybrids: Alta (Norway) × Fossbekk (Norway)	R. Lierelva	F	elimination in 2 weeks	innately resistant
Bakke *et al*. [26]	A: Lierelva (Norway)	R. Rauma	A	exponential growth	susceptible
	A: Lierelva and Batnfjordselva (Norway)	R. Batnfjordselva and Steinkjerselva	A and A*	exponential growth	susceptible
	A: Namsen and Alta (Norway)	R. Lierelva	F	exponential growth	susceptible
	A × B hybrids: Imsa (Norway) × Neva (Russia)	R. Lierelva	F	declining after 4 weeks	responding
	B: Neva (Russia)	R. Lierelva	F	declining after 3 weeks	responding
Bakke *et al*. [66]	A: Lierelva (Norway)	R. Figga	A*	exponential growth	susceptible
	B: Indalsälv (Sweden)	R. Figga	A*	slightly declining after 4 weeks	responding and susceptible
Cable *et al*. [42]	A: Alta and Lierelva (Norway)	R. Lierelva	F	exponential growth	susceptible
	B: Neva (Russia)	R. Lierelva	F	declining after 3 weeks	innately resistant and responding
Dalgaard *et al*. [38]	A: Conon (Scotland)	R. Lærdalselva	F	exponential growth	susceptible
	B: Lule (Sweden)	R. Lærdalselva	F	declining after 6 weeks	responding
Dalgaard *et al*. [39]	A: Conon (Scotland), Skjern (Denmark) and Bristol Cove (Canada)	R. Lærdalselva	F	exponential growth	susceptible
	B: Mörrum (Sweden)	R. Lærdalselva	F	exponential growth	susceptible
Jansen *et al*. [67]	A: Imsa (Norway)	R. Lierelva	F	exponential growth	susceptible
	♀A × ♂B hybrids: Imsa (Norway) × Neva (Russia)	R. Lierelva	F	exponential growth	susceptible
current study	A: Dee (Wales), Lærdalselva (Norway)	R. Fusta	A	exponential growth	susceptible

Footnotes: ^1^Host response presented using the three categories defined by Bakke *et al*. [26], *i.e.* susceptible, responding or innately resistant.

*Haplotypes tentatively proposed based on their geographic origin and their relative proximity to defined strains [4].

### The importance of including brown trout *Salmo trutta* in the current trial

One of the most interesting findings from the current trial arises from the infection of *G. salaris* on the population of *S. trutta* from the River Tyne. Prior to this study, *S. trutta* had been considered resistant to *G. salaris* infection. Jansen and Bakke [15], for example, infecting both individual and pooled (*i.e.* 50 fish per tank) samples of *S. trutta* with the strain of *G. salaris* from the River Lierelva (haplotype F), found that fish could carry an infection for up to 50 days. The current study found that when a pool of *S. trutta* were each given an initial infection of ~70 *G. salaris* per fish, then the *G. salaris* infections on these fish persisted for at least 110 days, when the experiment was terminated. Of these, seven of the 30 fish were still infected with between one and six parasites each.

*Salmo trutta* parr naturally infected with *G. salaris* at low intensities have been reported by a number of authors [68-71]. The studies by Tanum [68] and Mo [69] also demonstrated that *S. trutta* were able to maintain their *G. salaris* infections when cohabited with infected *S. salar*. A study by Bakke *et al*. [32] found that *S. trutta* exposed to infected fins of *S. salar* for 24 h and subsequently held in isolation eliminated their *G. salaris* infections in less than two weeks, suggesting that they could be innately resistant. Harris *et al*. [33] also considered *S. trutta* to be innately resistant to *G. salaris* when, after exposing groups of fish to infected *S. salar* fins for 24 h, the fish lost their infections within 42 days. In a survey by Jansen and Bakke [15], anadromous *S. trutta* from the River Lierelva were cohabited with heavily infected *S. salar* from the Lierelva for five days, and then either isolated and held individually or maintained as a group. In both cases, the infections of *G. salaris* on the *S. trutta* persisted for approximately 49 days p.i. In a repeat trial using a stock of *S. trutta* from Lake Tunhovd, Norway, the infection of *G. salaris* on the isolated *S. trutta* (n = 21) persisted for 28 days, whilst the infection on grouped fish (n = 21) lasted for 21 days p.i. These trials suggested that *S. trutta* can serve as a carrier for disseminating the parasite, although it is not able to support an infection with *G. salaris* for long periods [15].

The current study, however, found that English *S. trutta* can carry an infection of *G. salaris* for at least 110 days, and this finding appears to contradict those of previous studies [15,32,33]. This might be explained by genetic differences between each population of *S. trutta*, as also shown in a study [72] using three-spined sticklebacks, *Gasterosteus aculeatus* L., artificially infected with *Gyrodactylus gasterostei* Gläser, 1974, or by a potential different pathogenicity between *G. salaris* haplotypes (Table 2), *i.e.* Jansen and Bakke [15], Bakke *et al*. [32] and Harris *et al*. [33] who used haplotype F, while the current experiment used haplotype A. Additional studies, therefore, are required to elucidate this further.

### The importance of including grayling *Thymallus thymallus* in the current trial

English and Welsh grayling are commonly infected with *Gyrodactylus thymalli* Žitňan, 1960, a congener morphologically and genetically similar to or conspecific with *G. salaris* [60,61,67,73-75]. Previous experimental studies, however, suggested that the Lierelva strain of *G. salaris* (haplotype F) is unable to show high pathogenicity on the Scandinavian *T. thymallus*, although infections could persist for anything between 35 [16] and 143 days [34]. In both studies, the experiments were terminated with a low number of parasites still on their hosts. Likewise, the infections of *G. salaris* on *T. thymallus* in the current study were not completely outside the expected response, with a low level of parasites remaining on fish for the duration of the 110-day experiment. Only two out of the 30 *T. thymallus*, however, were still infected at the end of the trial. The finding that English *T. thymallus* can carry infections for long periods of time gives cause for concern in that they may play a role in extending the infection window for other more susceptible hosts. Given the debate regarding their conspecificity, that *G. thymalli* exists within the UK and that the UK has been separated from mainland Europe for ~200,000 years [37], the inclusion and experimental exposure of British *T. thymallus* to *G. salaris* was important.

### The experimental infection procedure

The period of experimental exposure used in the current study was 24 h and follows the methodology used in other *G. salaris* infection trials [16,32,42]. There is, however, no standard exposure period, and the times reported in the scientific literature appear to vary markedly, *e.g.* 48 h as used by Jansen *et al*. [67], Bakke *et al*. [66] and Dalgaard *et al*. [39], 72 h as employed by Bakke and MacKenzie [31], and up to two weeks in the study by Bakke *et al*. [30]. The experimental exposure period used in the current trial, however, was shown to be effective, resulting in a 100% prevalence of infection.

## Conclusions

The findings from this trial are significant in that they demonstrate: 1) that Welsh *S. salar*, as with Scottish *S. salar*, are also susceptible to *G. salaris*; 2) that *T. thymallus* respond in a similar manner to their Scandinavian counterparts and carry infections for up 110 days; and, 3) that English *S. trutta* are responsive to a *G. salaris* infection, but can harbour infections for longer than those reported for Norwegian populations, *i.e.* 110+ days as opposed to 50 days. The differences in *S. trutta* susceptibility observed in the present study and compared with previous Scandinavian trials [15,32,33], may suggest that potential genetic differences have been accumulated in *S. trutta* strains following their isolation since the last period of glaciation. These extended windows of infection and the interpretation of “resistance” need to be considered carefully in terms of the role that *S. trutta* could play within the context of national management planning and subsequent management in the event of a *G. salaris* outbreak.

Current national surveillance programmes for *G. salaris* in the UK focus on areas where *S. salar* are dominant, with relevant sites being sampled on a regular basis, *i.e.* at least once a year. Other sites, perhaps through limitations of manpower and other resources, are sampled less frequently. The demonstration from this study that *G. salaris* can persist on *S. trutta* for long periods would suggest that surveillance of *S. trutta* farms and of watercourses inhabited by *S. trutta*, especially where the two salmonids co-exist, should be increased. Given the suggested association of *O. mykiss* movements and emerging *G. salaris* infections, it is also recommended that during a suspected outbreak, *S. trutta* in and around *O. mykiss* sites are carefully monitored. While standard operating procedures (SOPs) for the processing and identification of *G. salaris* were recently addressed by Shinn *et al*. [76], based on the information from the present study, current national management plans, which already do not allow any fish movements in the event of a suspected outbreak, may benefit from a clarification of the potential role that *S. trutta* could play in the spread of *G. salaris*. The findings from this study demonstrate that *G. salaris* can persist on *S. trutta* for longer periods than previously thought and that reservoir hosts, such as *S. trutta* and *T. thymallus*, for *G. salaris* may play a more significant role in epidemics than previously believed.
